# Recent Progress in Structural Integrity Evaluation of Microelectronic Packaging Using Scanning Acoustic Microscopy (SAM): A Review

**DOI:** 10.3390/s25247499

**Published:** 2025-12-10

**Authors:** Pouria Meshki Zadeh, Sebastian Brand, Ehsan Dehghan-Niri

**Affiliations:** 1 School of Manufacturing Systems and Networks, Ira A. Fulton Schools of Engineering, Arizona State University, Mesa, AZ 85212, USA; 2 Fraunhofer Institute for Microstructure of Materials and Systems IMWS, Walter-Hülse-St., 06120 Halle, Germany

**Keywords:** microelectronic packaging, semiconductors, nondestructive evaluation, heterogeneous packages, 3D-ICs, artificial intelligence, Scanning Acoustic Microscope

## Abstract

Microelectronic packaging is crucial for protecting, powering, and interconnecting semiconductor chips, playing a critical role in the functionality and reliability of electronic devices. With the growth in complexity and miniaturization of these products, the implementation of efficient inspection techniques becomes crucial in preventing failures that may result in device malfunctions. This review paper examines the progress made in utilizing Scanning Acoustic Microscopy (SAM) to assess the structural integrity of microelectronic systems within the broader field of Nondestructive Evaluation/Testing (NDE/T) methods. With an exclusive emphasis on SAM, we point out SAM technological advancements in multi-die stacking, Through Silicon Vias (TSV), and hybrid bonding inspection that improve inspection sensitivity and resolution required to be prepared for upcoming challenges accompanying 3D- and heterogeneous integration architectures. Some of these approaches compromise the depth of inspection for the benefit of lateral resolution, while others do not sacrifice the in-depth range of evaluation. These developments are of the utmost importance in addressing the substantial obstacles associated with examining microelectronic packages, facilitating the early detection of potential failures, and enhancing the reliability and robustness of semiconductor devices. Furthermore, our discussion consists of the fundamental principles and practical approaches of SAM. It also examines recent investigations that integrate SAM with machine learning concepts and the application of deep learning models in order to automate defect detection and characterization, thus substantially augmenting the efficiency of microelectronic package assessments.

## 1. Introduction

**Figure 1:**
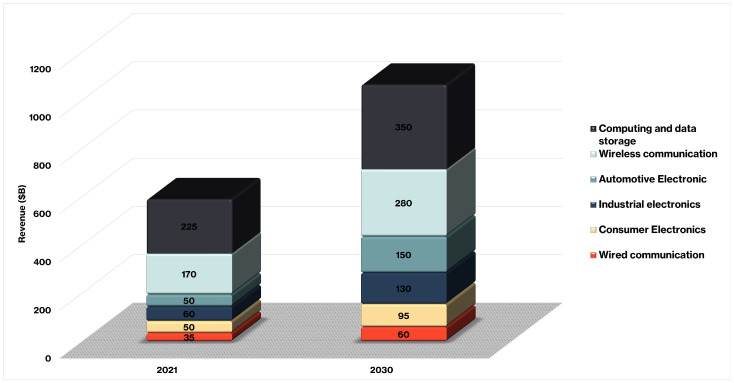
Estimation of microelectronic packaging market size from 2020 to 2030: A comparative analysis of key industries [6].

The field of semiconductor device technology has had significant advancements since the introduction of the transistor in 1947 and the invention of the Integrated Circuit (IC) in 1958 [1]. This expansion encompasses the density, intricacy, and market utilization of devices [2,3]. Currently, semiconductors are essential elements in a wide range of electronic products, including smartphones, personal computers, industrial robots, autonomous cars, and others [4,5]. Published reports confirm the critical significance of ICs and semiconductor devices. They revealed the market size of microelectronic packaging production in some industries, which was estimated to be around $590B (see Figure 1). The prediction estimates that the market size will grow to $1065B by 2030.

**Figure 2:**
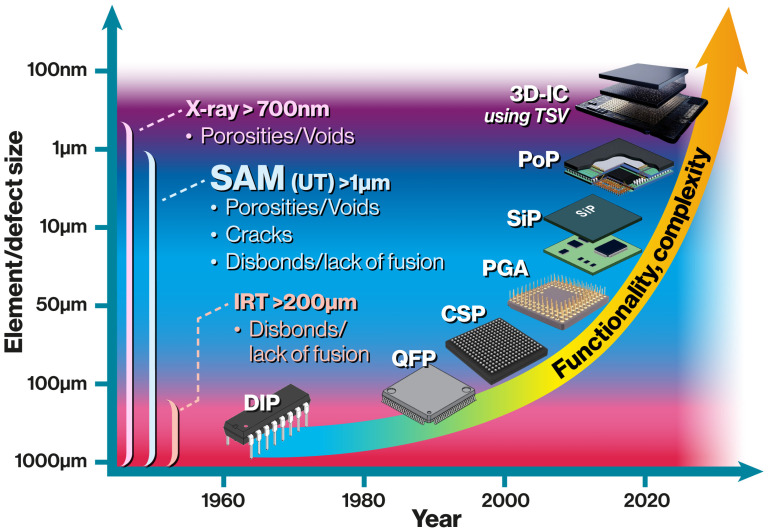
Evolution of microelectronic packages.

The community employs the term *“Advanced packages”* to refer to new designs where the number of Input/Output (I/O) pins has increased and the size has shrunk. In parallel with the traditional Moore’s Law, which has been dictating the pace of scaling for logic devices, packaging is currently witnessing impressive growth with an exponential increase in I/O density [7]. According to this law, the number of transistors per chip doubles every two years [8,9]. As electronic devices become smaller, lighter, and more energy-efficient, there is a growing demand for the semiconductor industry to innovate new integration concepts and packaging techniques that are compact and capable of meeting computing demands [10]. In Figure 2, it can be seen that the number of I/O pins, size, and the number of ICs in a single package have increased with new designs surpassing the previous models.

Manufacturing IC packages involves several crucial steps that can impact their performance [11]. With the current trend of increasing the number of production steps and the miniaturization of components, there is a probability of a rise in manufacturing defects within such packages.

Monitoring the manufacturing process of microelectronic packages and performing thorough inspections after each production step are therefore essential for ensuring product quality and reliability [12]. Advanced monitoring and complementary inspection approaches are required since not all defects can be detected through standard electrical testing alone [13]. These evaluation steps typically rely on either destructive or nondestructive testing (NDT) methods. Common destructive techniques, such as cross-sectioning and dye-and-pry analysis, can reveal internal defects but irreversibly alter the specimen [14,15,16,17]. In contrast, nondestructive approaches assess the structural integrity of components without causing damage. Widely used NDT methods include X-ray imaging, infrared thermography (IRT), and various ultrasonic-based techniques.

Conventional NDE/T methods developed for bulk or homogeneous materials cannot be directly applied to the inspection of microelectronic packages. These packages consist of multiple ultra-thin layers, semiconductor dies, metallic interconnects, polymer-based underfills, and plastic encapsulants, with widely varying acoustic properties and micrometer-scale thicknesses. This heterogeneity leads to strong internal reflections, acoustic scattering, and signal attenuation, which complicate defect localization and quantitative interpretation, thereby making traditional NDE/T inspection and analysis challenging. Moreover, many defects of interest, such as delamination at die-attach interfaces, voids within underfill, or cracks along Cu–Cu bonding layers, are interfacial and submicron in scale, demanding far higher spatial resolution and sensitivity than those typically used in conventional NDE/T applications. Consequently, the evaluation of microelectronic packaging constitutes a specialized task within the broader field of nondestructive inspection, requiring adapted techniques, tailored instrumentation, and advanced signal processing methodologies.

One effective contribution to these demands can be provided by Scanning Acoustic Microscopy (SAM), an immersion-based ultrasonic testing (UT) technique widely adopted in the microelectronic packaging and manufacturing industry. Immersion UT is broadly used for evaluating composite and metallic structures across various sectors; however, what distinguishes SAM is its combination of high-frequency ultrasound, strongly focused acoustic lenses with large numerical apertures and precise mechanical scanning, which enables visualization of internal interfaces and defects with micrometer-scale resolution. Alongside SAM, more advanced inspection systems such as high-power X-ray computed tomography (XCT) and modulated IRT have been developed to address the increasing complexity of modern microelectronic packages.

Since each technique provides complementary capabilities and possesses inherent limitations, as illustrated in Figure 2, the fusion of multiple NDE/T methods at different production stages offers a robust inspection strategy for meeting emerging challenges [18]. For example, IRT can be rapid test for revealing disbonds, voids, and delamination, while SAM is more time-consuming but provides detailed interfacial imaging that precisely localizes these discontinuities. Likewise, combining SAM with X-ray computed tomography (XCT) merges acoustic and density contrast information, allowing for three-dimensional mapping of internal structures and improved distinction between fine volumetric and planar discontinuities. Such multi-modal approaches enhance the reliability and comprehensiveness of nondestructive assessments in advanced packaging.

Several researchers have published a review examining the applications of various NDT methods for specific types of packaging. A review of the inspection of several IC packages was given by Aryan et al. [19]. They discussed examples of using X-ray, IRT, infrared microscopy, magnetic current reflectometry, ultrafast optical laser inspection, SAM, and Surface Acoustic Waves. In 2020, Su et al. [20] conducted an in-depth review of research that employed Optical methods, XCT, Thermography, SAM, and Laser vibration techniques to identify anomalies in packages with solder connections. A recent publication by another research group examined the current state and advancements in the nondestructive evaluation of microelectronic packages. This review also included investigations on hybrid ultrasonic-laser digital holography microscopy [21]. Varshney et al. [22] discussed the limitations and challenges of applying nondestructive evaluation methods, particularly SAM and 3D X-ray imaging, for reliability assessment of 2.5D and 3D heterogeneous integrated packages. The paper highlights technological gaps in resolution, signal-to-noise ratio, and data interpretation that hinder the effective localization of defects in stacked dies and dense interconnect structures.

Considering these unique characteristics of microelectronic packaging and the versatility of ultrasonic inspection, this contribution is dedicated to research on the integrity assessment of semiconductor and microelectronic packaging using SAM. Despite certain limitations, SAM remains a powerful nondestructive technique for examining complex, multilayered packages and can effectively complement other NDT methods. The novelty of this review lies in its systematic classification of SAM studies according to interconnection and structural categories, its evaluation of SAM applications in the inspection of advanced and heterogeneous packaging (e.g., TSVs and Cu–Cu bonding), and its synthesis of recent developments in artificial intelligence and machine learning for automated damage detection and material characterization. Furthermore, techniques aimed at enhancing image quality and improving flaw detectability through advanced signal and image processing are discussed in detail.

The structure of this article is organized as follows. Section 2 introduces the fundamental packaging types and highlights potential factors contributing to defect initiation and propagation. This is followed by a concise overview of existing inspection approaches employing X-ray, thermography, and conventional ultrasonic testing. Section 3 outlines the principles and fundamentals of SAM as an immersion-based ultrasonic inspection method. Section 4 is divided into three subsections: Section 4.1 summarizes SAM applications for defect identification, categorized by the primary interface or structural region where flaws typically originate, ranging from die-attach and sintered layers to bump/underfill zones, wire-bond and mold/leadframe interfaces, TSVs/interposers, and Cu–Cu bonded structures; Section 4.2 reviews methodologies for enhancing image resolution and diagnostic sensitivity; and Section 4.3 surveys efforts toward automation through data-driven and machine-learning-based approaches. Finally, Section 5 consolidates key findings and proposes emerging research directions for advancing the use of SAM in microelectronic inspection.

## 2. Microelectronic Packaging and Inspection

### 2.1. Packaging and Defect Formation

Originally, an electronic package can be described as an enclosure with interconnections for chips, designed to fulfill four key functions:

- Provide structural support for the chip;
- Protect the chip from environmental effects;
- Facilitate effective heat dissipation produced by the chips;
- Enable electrical connectivity for power and signal transfer.

The significance of these functions and potential defects that may arise due to the package’s inadequate performance in fulfilling these objectives is discussed in the sections that follow.

#### 2.1.1. Environmental Protection

ICs require protection from their environment primarily due to the presence of moisture, which can lead to and accelerate chemical reactions, such as corrosion of the internal components housed within the packages. In addition, good packaging can eliminate potential issues such as particles and ionic contaminants, which may compromise the lifespan and reliability of the IC. Sealing and encapsulation are two approaches used to protect chips and electrical components from environmental hazards.

#### 2.1.2. Mechanical Protection and Thermal Management

The manufacturing process of electrical components involves multiple stages. Throughout this procedure, the components will be subjected to temperature variations, vibrations, and physical stresses. Thus, the mechanical strength and stability of these components are crucial in preserving the dies and internal structures. To accomplish this, die attachment techniques are used to secure the die in a stable position and preserve the integrity of the interior structure. Typical methods employed by microelectronic packaging companies to ensure secure attachment of the die to a substrate include:

- Eutectic bonding;
- Soldering;
- Epoxy;
- Resins;
- Sintering (Ag, Cu).

Epoxies are a cost-effective method for die attachment but may not provide the best thermal management, making them less desirable for applications that require high thermal conductivity of the die-attach. Additionally, their vulnerability to delamination and the potential impact of foreign inclusions on their mechanical properties limit their use in specific scenarios. Soldering presents a better option for thermal management, particularly with solder bumps and eutectic bonds offering higher thermal conductivity. However, the viability of solder joints for high-temperature applications becomes compromised at temperatures above 200 °C due to challenges such as the emission of hazardous substances from Pb-based solders and the diminished fatigue strength resulting from Cu-Sn Intermetallic Compounds (IMCs) [23,24,25,26,27]. Sintering with silver-glass composites is favored for high-temperature applications, such as power modules, due to its superior thermal stability, conductivity, and enhanced electrical and mechanical characteristics [28]. Yet, sintered layers face challenges, including susceptibility to pore formation, which can affect the bond’s mechanical and thermal properties and connectivity over time.

#### 2.1.3. Electrical Connection

One of the most fundamental functions of a microelectronic package is to establish reliable electrical connections among its constituent elements. In conventional designs, these connections are realized at discrete hierarchical levels, but in modern 3D and heterogeneous systems, the distinction between chip-level, package-level, and board-level interconnections has become increasingly blurred. A unified view of the interconnection hierarchy is therefore necessary to capture the electrical and mechanical coupling across scales. The primary interconnection levels can be categorized as follows:

- *Chip-level interconnections*: These reside within the back-end-of-line (BEOL) of the silicon die and serve as microscopic wiring networks distributing power and signals among transistors and circuit blocks. They consist of alternating metal and dielectric layers (typically Cu or AlCu with SiO_2_ or low-*k* dielectrics) and terminate at the topmost redistribution layers that interface with the first-level interconnects.
- *First-level interconnections*: Often referred to as *package-level* interconnections, these connect the active dies to a package substrate or interposer. Common implementations include wire bonding (WB), flip-chip solder bumps, and hybrid Cu-to-Cu bonding. Certain vertical interconnects, such as through-silicon vias (TSVs), may also fall within this category when fabricated in passive interposers, though in stacked-die architectures, TSVs can instead function as chip-level pathways linking multiple active dies.
- *Second-level interconnections*: Sometimes referred to as *board-level* connections, these bridge the package substrate to the printed circuit board (PCB) through solder balls, pins, or other surface-mount contacts. They enable communication and power delivery between packaged devices and the larger electronic system.

Advances in 3D and heterogeneous integration have led to tighter coupling between all interconnection levels. Vertical stacking, redistribution layers, and through-silicon structures have merged on-die metallization with package and board interfaces, creating a continuous network of conductive pathways across the entire electronic assembly. As a result, many reliability mechanisms, such as electromigration, delamination, IMC growth, and void formation, now manifest collectively throughout this hierarchy rather than at isolated junctions.

Various interconnection schemes are employed across microelectronic packaging hierarchies. Traditional package-level interconnections include Tape Automated Bonding (TAB) and G-shaped springs, whereas on-die interconnections comprise thin-film metallization and AlCu wiring within the back-end-of-line (BEOL). Each of these connection types was developed to address specific design, density, or thermal-performance requirements and shares similar reliability concerns with the more common interconnection methods [29,30,31,32].

Four electrical connection techniques are particularly emphasized in the literature and are frequently investigated in structural assessment studies: Wire Bonding (WB), solder-based joining, Through-Silicon Vias (TSVs), and Cu-to-Cu (direct or hybrid) bonding. Therefore, further details are provided on the configuration, reliability mechanisms, and typical defect types associated with each of these interconnections.

**Figure 3:**
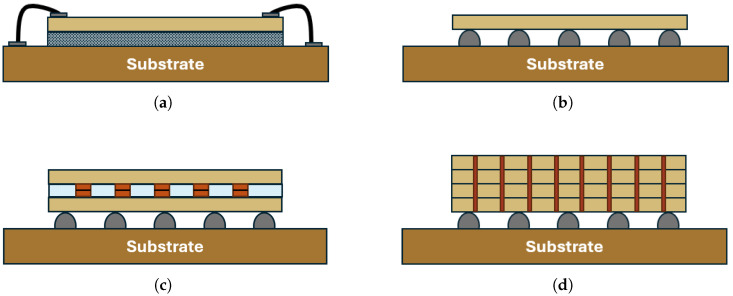
Examples of common interconnection techniques in microelectronic packages: (**a**) Wire Bonding; (**b**) Flip-Chip; (**c**) Hybrid Bonding; (**d**) Through Silicon Vias.

**Wire bonding**, recognized as the industry’s oldest method for chip-package bonding [33,34], relies on the physical attachment of a chip’s backside to the substrate, with wires connecting to conductive bond pads on the top side to establish circuitry electrical connections, as shown in Figure 3a. Wedge bonding employs pressure and ultrasonic vibration, operating at low temperatures with vibrations ranging from 20 to 300 kHz [2]. Conversely, Ball bonding typically uses thermocompression or thermosonic processes. Interested readers are encouraged to go through the content of [2] for further information on ball/wedge bonding and the associated connection method.

WBs are commonly made of gold (Au), copper (Cu), or silver (Ag), while aluminum is usually used for the bond pad material. When high-temperature bonding methods like thermocompression and thermosonic methods are used, the differences in materials can lead to the formation of IMCs [35]. While the creation of IMCs is generally considered beneficial as it indicates the quality of bonding, it can also lead to the formation of flaws. When gold wires are connected to aluminum pads, the disparities in atomic diffusion rates can cause Kirkendall voids, which can result in the ball lifting off the chip and ultimately leading to bonding failure [36,37]. The higher resistance of IMCs compared to pure metals, can result in additional problems [38]. Electromigration (EM), a phenomenon seen in wire bonding, entails the migration of atoms within a conductor at high current densities. During EM, voids can form at the cathode, and hillocks can form at the anode. High current densities can also exacerbate IMC growth, which in turn leads to localized Joule heating effects, further compounding the problem.

**Solder-based interconnections** represent one of the most versatile and widely used methods for establishing electrical and mechanical continuity in microelectronic assemblies. They are employed across multiple integration scales—from connecting dies to substrates at the package level to mounting entire packages onto printed circuit boards (PCBs). These joints rely on metallic alloys that reflow to form conductive bonds between metallized pads on opposing components.

At smaller scales, solder bumps or micro-bumps are used to connect the die to the substrate or interposer, allowing input/output (I/O) terminals to be distributed across the die surface rather than confined to its periphery, Figure 3b. This approach, commonly known as wafer-level assembly, requires the deposition of solder bumps on the wafer prior to dicing [34,39]. To ensure adequate adhesion and electrical contact, the wafer surface is first cleaned and roughened, followed by the sequential deposition of metallic layers that form a wettable interface with low contact resistance—commonly referred to as Under Bump Metallization (UBM) [40]. Depending on the specific application, bonding between solder and substrate pads can be achieved through thermocompression, ultrasonic, or thermosonic techniques, comparable to those utilized in wire bonding processes.

Despite their broad applicability, solder joints remain susceptible to several reliability concerns. Thermal fatigue and electromigration are among the most prominent degradation mechanisms. Differences in the coefficients of thermal expansion (CTE) between the solder and the adjoining materials induce cyclic mechanical stresses, leading to the formation of cracks and interfacial damage over time [41,42]. Elevated temperatures accelerate microstructural evolution within the solder, reducing its fatigue resistance [43,44]. Under high current densities, electromigration drives the directional diffusion of metal atoms, which can cause void formation at the cathode and hillock growth at the anode, further exacerbated by Joule heating effects. Solder joint fatigue failure typically manifests in three modes: Mode *I*, the appearance of cracks at the interface between the intermetallic compound (IMC) and the solder on the PCB side; Mode *II*, cracking within the solder/IMC interface; and Mode *III*, separation of the solder from the chip or pad due to copper dissolution within the UBM layer [20].

In more severe cases, solder bumps may detach entirely, disrupting electrical connectivity between the die and substrate. The use of underfill materials is a well-established mitigation strategy to reduce thermal mismatch, distribute mechanical stress, and enhance the long-term fatigue resistance of solder-based assemblies [45,46].

**TSVs** are etched holes that pass through the silicon wafer, as shown in Figure 3d, whose surfaces are coated with insulating materials before being filled with copper. Together with hybrid Cu-to-Cu bonding, they represent a major step toward meeting the ever-increasing demand for higher interconnect density and faster signal transmission in advanced electronic systems. These technologies enable vertical stacking and integration of multiple ICs, facilitating the creation of heterogeneous packages and true three-dimensional architectures.

Although the fabrication of TSVs is highly controlled, several manufacturing challenges can still arise, such as void formation during copper filling. Tapered or rough sidewalls generated in the etching process can hinder uniform deposition of both the insulation line and the filling material, and the high aspect ratio of TSV holes can further promote incomplete filling. In service, electromigration may occur, particularly when smaller solder bumps are used and a larger number of dies are stacked, accelerating void growth and local resistance increases.

**Cu-to-Cu bonding** is employed to construct multilayer or stacked packages and can function either alongside TSVs or as a stand-alone interconnection method. This technology encompasses two main approaches: thermocompression bonding and hybrid (oxide–Cu) bonding [47]. Thermocompression bonding relies on the application of high temperature and pressure, requiring specialized chambers and extended process times. In contrast, hybrid bonding operates at lower temperatures and enables interconnection pitches below 10 μm [48]. It establishes a permanent connection that combines a dielectric bond with an embedded metallic (usually Cu) bond. Figure 3c illustrates a schematic of such Cu-based joints.

Since the dielectric bonds form at ambient temperature, elevated pressure is not required, simplifying subsequent processing. A two-phase annealing sequence follows: the first stage strengthens the dielectric bonds, and the second promotes interdiffusion of the copper pads [49]. Key process factors include achieving sub-nanometer surface roughness, maintaining low contamination levels, and ensuring precise pad alignment. Excessive copper protrusion during annealing can create gaps between dielectric layers, while excessive dishing may lead to voids due to insufficient Cu–Cu contact [50].

**Figure 4:**
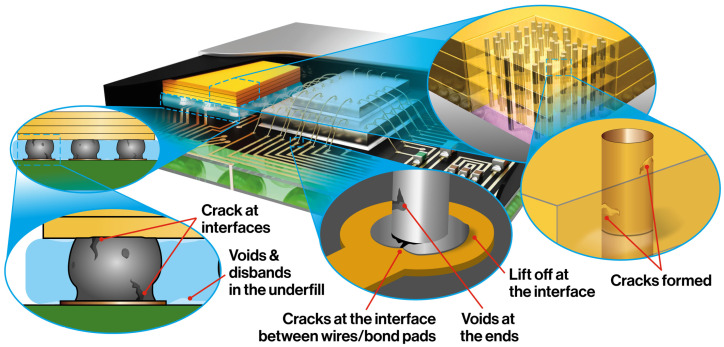
Representative defects observed in integrated circuit (IC) packages.

To encapsulate the range of defect types and their locations within microelectronic interconnections, a System-in-Package (SiP) incorporating multiple interconnection types and associated flaws is shown in Figure 4. This illustration highlights the diversity of interconnection technologies used in modern electronic packaging and identifies common defect sites, providing a concise overview of regions most susceptible to failure across different interconnection schemes.

### 2.2. NDE Methods for Microelectronic Packaging

In addition to SAM, three other prevalent NDE/T methods mentioned in recent literature are X-ray, Infrared IRT, and UT. This section will provide an overview of the various applications of these methodologies in the context of identifying damage in microelectronic systems.

#### 2.2.1. Radiography

**Figure 5:**
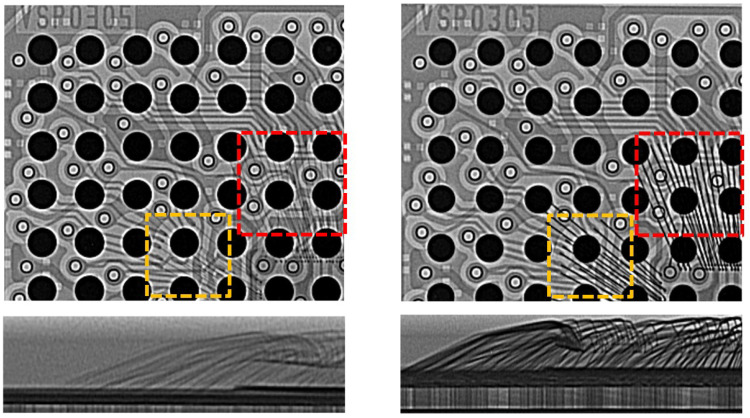
An X-ray image displaying a counterfeit within a Ball Grid Array (BGA) packaging. The genuine gold bonding wire is shown on the right side, and a counterfeit copper one is on the left [60]. The two dashed boxes refer to similar areas within the two samples for comparison.

X-ray images are generated as a result of recording the penetrated beams into the material and providing a map of internal structures [51,52]. 2D radiography and XCT are the most common X-ray testing approaches. 2D imaging, which is more conventional for different inspection purposes, can identify defects in electronic microsystems [53]; nonetheless, the technique cannot fulfill the requirement for inspecting fine and complex structures such as 3D-ICs. XCT, on the other hand, enables the examination of a sample from many viewing angles achieved through sample rotation, offering a more comprehensive and detailed analysis. Consequently, it is particularly suitable for the inspection of intricate designs. Such merits of 3D X-ray test have been exploited in a variety of studies [54,55,56,57]. Different failure mechanisms caused by electromigration in micro-solder bumps were studied by Shie et al. [58] using 3D X-ray. Counterfeits are another type of defect investigated by X-ray technology. Such a category of defects is defined as any deviation in the internal structure of packages, bond wire types, die dimensions, etc. with respect to a reference sample [59]. The Figure 5 shows an example of such anomalies.

**Figure 6:**
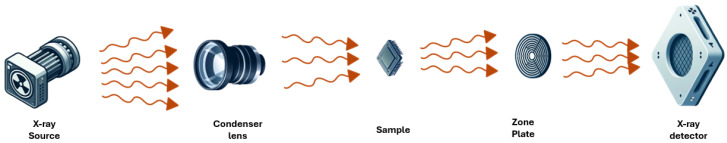
XRM imaging architecture in three dimensions using Fresnel zone plate targets.

Muß and Koch [60] discussed the challenges in the identification of counterfeits based on X-ray radiography images and presented a rapid solution to inspect wire bonds in electronic packages. To improve the resolution of X-ray images, the principle of geometric magnification is commonly used, which relies on changing the distance between the sample and the source. The process, so called micro-XCT, suffers limitations, including being unable to capture the critical discontinuities in miniaturized 3D packages [61]. Optical magnification can be combined with geometrical magnification, which is the basis of X-ray microscopy (XRM). XRM provides high-resolution images even when the sample is placed relatively far from the source [62]. Figure 6 shows the schematic of a 3D nanoscale XRM architecture.

The further improvement in resolution and contrast of XRM images can provide more detailed information on anomalies within micro-packages. This has been the topic of the Gu et al. [63] study, where they proposed a new scintillator material coupled with the objective lens to improve the quality of images and reduce computational costs. With all the merits of X-ray imaging, the method has some major drawbacks, such as the substantial time required for inspection. Mohammad-Zulkifli et al. [64] employed XRM to detect C4 bumps, and they reported that the scan time of an area with 2.8 mm in diameter was about 30 min. Another issue is that advanced IC packages are susceptible to damage due to high-power X-ray beams and long scans [65,66].

#### 2.2.2. Infrared Thermography (IRT)

Thermography is a widely applied NDT technique across industries such as aerospace, automotive, oil and gas, and microelectronics. The method relies on detecting temperature variations that arise between intact and defective regions. In microelectronic packaging, active IRT is typically employed, in which an external heat source induces a transient thermal response. Because excessive heating can alter or damage sensitive devices, the excitation is kept low, producing only slight temperature gradients while maintaining full functionality of the package. This inherent non-invasive characteristic provides IRT with an advantage over X-ray methods, whose ionizing radiation can be harmful to components or operators. Moreover, IRT offers rapid data acquisition and is well suited for inline inspection of microelectronic systems [67,68]. Recent studies have therefore focused on leveraging IRT for detecting various flaws in integrated-circuit (IC) packages. These include delamination in sintered parts, voids and foreign objects in molded packages, and flaws in solder-based packages [69,70,71,72,73]. In addition, IRT serves as a practical instrument for predicting the lifespan and assessing the reliability of microelectronic systems [74,75]. Lyons et al. [76] investigated the concurrent utilization of IRT and Digital Image Correlation (DIC) to assess the reliability of semiconductor packages subjected to thermomechanical stresses. They suggested a one-shot strategy to address the variation in sample preparation needs between IRT and DIC, which hindered the concurrent use of these techniques. Zhou et al. employed eddy current pulses (ECPT) as a means to stimulate BGA Packages in their study [77]. The objective of their research was to determine the remaining lifespan of solders at the substrate/PCB interface using numerical and experimental investigations. The thermal cycling necessary for the ECPT measurement was achieved by simulating the working environment using a heating source. The Darveaux model was employed to estimate the remaining life of the package. This model relies on estimating the time needed for crack formation by considering the average dissipated energy density and the crack length when the solder fails, based on principles of fracture mechanics.

The signal-to-noise ratio (SNR) and spatial resolution of thermal images have long been primary concerns in infrared thermography. Researchers have addressed these challenges mainly by refining the heating regime and applying advanced signal processing techniques. One particularly effective approach involves using a modulated heating regime, in which the excitation is periodically varied, most commonly in a sinusoidal form. This principle led to the emergence of a dedicated branch of thermographic inspection known as lock-in thermography (LIT), which enhances defect detectability by synchronizing the thermal excitation and image acquisition phases [78].

Panahandeh et al. [79] proposed the utilization of LIT, a technique that depends on the production of sinusoidal heat pulses. Their research shows that utilizing modulated pulses analyzed using the Fourier transform can effectively detect thermal contrast below the noise threshold of infrared camera systems. Brand et al. [80] attempted to improve the three-dimensional Lock-In Thermography (3D-LIT) results by spatial phase evolution. By analyzing the lateral phase distribution and reconstructing the thermal wavefront, they effectively compensated for diffusion-related blurring and localized thermally active defects in both lateral and depth directions with micrometer-scale precision. This technique enables nondestructive 3D defect localization in complex stacked-die microelectronic packages, demonstrating LIT’s growing potential as a quantitative inspection tool for next-generation semiconductor devices. A different study employed Barker Code Infrared Thermography (BCIT) and Linear Frequency Modulation Thermal Wave Imaging (LFMTWI) methods to identify micro-crack flaws in silicon wafers. The performance of these techniques was evaluated by comparing their ability to enhance the SNR improvement [81].

IRT inspection is also accompanied by some limitations. Arguably, the most prominent issue is the challenge of obtaining the location of defects, because of the intricate structure of micropackages and the fact that thermal cameras record sample surface temperatures, faults at varying depths may exhibit comparable thermal traces. Furthermore, IRT tests may necessitate certain preprocessing measures to maximize the test’s efficiency.

#### 2.2.3. Low Frequency Ultrasonic Testing (UT)

Another extensively employed NDE/T method is Ultrasound testing. UT fundamentally relies on the interaction of mechanical waves generated by transducers with anomalies within the materials, leading to a reduction in the amplitude of the received signal, the generation of higher harmonics, or an alteration in the phase-space representation of the system state. The application of this method in electronic packaging inspection can be classified based on the excitation approach.

The generation of surface waves, a group of ultrasonic waves, with contact transducers and finding delamination defects within IC packages have been investigated by many researchers. The research by Li et al. [82] looked into how to find delamination in the sealant area where the heat sink is attached to the substrate. In their contribution, the time-of-flight (ToF) and amplitude of the wave modes were two features that fed a multivariate Gaussian model to detect delamination. The same research group investigated the impact of preprocessing by applying band-pass filtering and cross-talk elimination [83]. ToF and the amplitude of the first-arrived packet were the inputs of the density-based spatial clustering of applications with noise (DBSCAN).

Laser is another source used for UT micro-packages inspection. In the Laser Ultrasonic (LUT) setup, a laser interferometer is employed to quantify the out-of-displacement signal, which serves as an indicator of the magnitude of vibrations occurring on the package’s surface. The defect or failure estimation is performed using a statistical comparison approach, whereby the interferometer signal from the test vehicle is compared with that of a known excellent reference sample [84]. LUT has been used to detect missing solder bumps, cracks, non-wetting defects in solder bumps, voids, and micro-cracks in solder balls of Plastic BGA packages, estimating the remaining life of such packages, as well as void detection with heterogeneous packages [85,86,87,88]. Mebane et al. [89] used an optical fiber to form the laser beam on the sample. They investigated the application of dual fibers compared to single fibers with respect to peak-to-peak energy and SNR wave responses. According to their findings, cracks with a length of 5 μm could be detected in FCBGA packages. Mehendale et al. [90] used short laser pulses, excited for 300 ps, to find artificial voids formed in Cu interconnections of 3D-ICs. The sizes of Cu fills were 5 × 50 and 10 × 100 μm. The same research group also investigated void detection in 3 × 50 μm vias [91]. They reported that near-surface voids as small as 0.4 μm could have been detected.

## 3. SAM Principle

**Figure 7:**
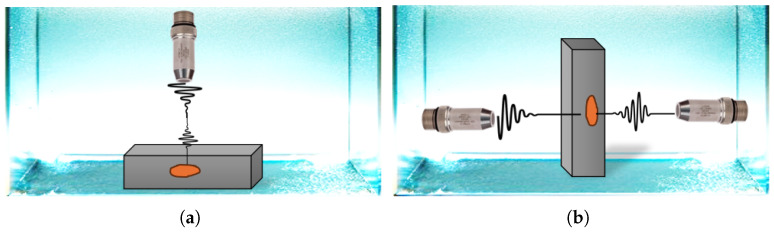
Different ultrasonic testing (UT) configurations: (**a**) Pulse–Echo, where a single transducer acts as both transmitter and receiver; and (**b**) Through–Transmission, where separate transducers are used for emission and detection.

The SAM technique, a UT test variation, utilizes mechanical waves to detect discontinuities in samples. This is an immersion technique in which water functions as the couplant, an essential requirement for most UT investigations. Acoustic waves are produced using focused apertures or flat transducers and travel through water and into the materials under inspection. At each interface, materials of differing mechanical or elastic properties result in a portion of the incident acoustic waves being reflected. The same transducer that has generated the acoustic signals will then receive the reflected and backscattered waves. The term “pulse-echo” describes this setup, in which a single transducer serves as both the transmitter and the receiver. Alternatively, if distinct transducers are employed for the sender and the receiver, the arrangement will be referred to as pitch-catch or through-transmission, depending on the positioning of the transducers. With regard to immersion UT configurations, the pulse-echo and through-transmission methods are the more conventional arrangements. Figure 7 provides a schematic illustration of these configurations as commonly implemented in immersion UT systems.

**Figure 8:**
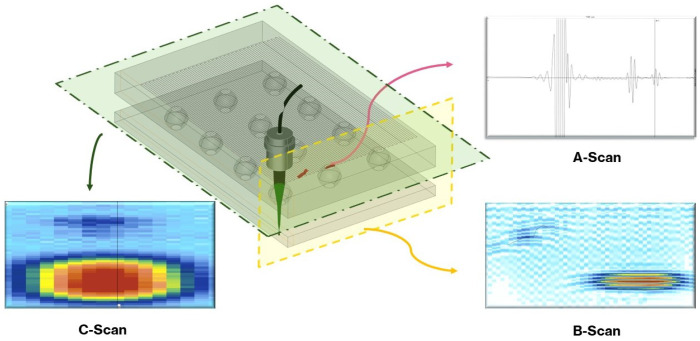
Schematic representations of UT results: the A-scan shows signal amplitude versus time-of-flight; the B-scan compiles A-scans along a line to provide depth-resolved information; and the C-scan maps the gated amplitude over a 2D raster at a selected time-of-flight window (time-gate) corresponding to a specific interface within the multilayer structure.

The interaction between mechanical waves and various materials in the structure under investigation can impact the reflected waveform, which serves as a means of detecting anomalies, as it is represented in Figure 8. The signal received by the transducer at a single location is referred to as an A-scan, as it reflects the amplitudes versus time of flight. When recording A-scans along a scan line in the sample, the amplitudes are commonly rectified, low-pass filtered, and gray-scaled and represented as a 2D image. The representation is referred to as a B-scan, as the amplitudes are represented through the brightness.

The third representation approach, which is most used in microelectronic failure analysis, is the C-scan. It represents the amplitudes of the acoustic signal within a defined time gate, recorded in a 2D raster scan across the sample. The time gate defines a specific time window on the A-scan, characterized by a start time, duration, and amplitude threshold, that corresponds to a particular depth or interface within the multilayered structure. In practice, the gate is positioned to isolate the portion of the reflected waveform originating from a specific boundary (e.g., die-attach, solder bump, or mold interface). Depending on the gate definition, the signal can represent either the maximum amplitude within the gate or the first threshold crossing event that occurs within the gate. By scanning over the entire region and extracting the gated amplitude from each A-scan, a single C-scan image is produced, mapping reflectivity or defect distribution at that selected depth. Sequentially shifting the gate through the waveforms enables the reconstruction of multiple C-scans at different interfaces, effectively allowing for three-dimensional visualization of internal features. Consequently, while the B-scan provides in-depth variations along a single line, the C-scan offers lateral information at a fixed depth, as illustrated in Figure 8.

The clarity of UT data is intrinsically linked to the frequency of the emitted pulses, meaning that the capability to identify smaller defects increases with a rise in the frequency of the initial pulses. In fact, the frequency of the generated wave correlates with the wavelength within a material through the well-known relationship λ=v/f, where λ and *v* denote the propagated wavelength and the speed of sound within the material, respectively. The sensitivity of ultrasonic waves to discontinuities is commonly characterized by the ratio of the defect size to the wavelength. As a practical rule of thumb, when the defect dimension (*d*) exceeds roughly half of the wavelength (i.e., when λ/d<2), the defect becomes highly likely to be detected.

Previous studies leveraging SAM for inspecting micro-packages have utilized frequencies between 5 and 50 MHz [20]. However, with the continual reduction in the size of modern package features, it has become necessary to employ higher frequencies to enhance data precision and mitigate the risk of flaws and connection overlap within tightly packed microelectronic systems. Consequently, recent advancements have shifted toward employing significantly higher frequency waves, spanning from 110 MHz to 1 GHz, allowing for the generation of ultrasonic wavelengths in the micrometer to sub-micrometer range. This progression enables finer spatial resolution, which is crucial for pinpointing defects in the compact configurations of modern microelectronics.

Despite its established role in inspection of semiconductor packages, which is discussed in detail in Section 4.1, SAM exhibits several inherent limitations that must be considered. The foremost constraint is the trade-off between inspection depth and the ability to resolve fine features. Increasing the excitation frequency enables the detection of minute defects and improves the separation of neighboring interfaces, allowing for the generation of distinct C-scans. However, the higher frequency also intensifies acoustic attenuation within the sample, restricting the penetration depth. The use of a liquid couplant, typically water, is another unavoidable limitation that restricts the inspection of moisture-sensitive components and complicates inline testing. In addition, complex multi-interface structures can produce overlapping echoes, including reflections from the transducer lens, which complicates the isolation of individual interfaces and the definition of accurate time gates for C-scan reconstruction. High-frequency operation results in large data volumes and intensive computational requirements due to the higher sampling rates. Moreover, the optimal focus depth often varies across the sample, necessitating multiple scans at different focal positions to achieve uniform image sharpness.

Recent advances discussed in Section 4.2 and Section 4.3 offer partial solutions to these challenges. GHz-SAM improves feature discrimination and reduces signal overlap between adjacent layers, although it remains unsuitable for highly attenuative materials and shallow penetration depths. Signal- and image-processing algorithms (Section 4.2) can enhance apparent resolution without increasing frequency, thereby limiting data volume and compensating for image degradation in slightly defocused regions. Furthermore, machine- and deep-learning techniques (Section 4.3) have shown promise in improving defect interpretation by directly extracting latent features from A-scan signals, enabling the identification of anomalies that may not be visually separable in conventional C-scan images.

## 4. Integrity Assessment Using SAM

**Figure 9:**
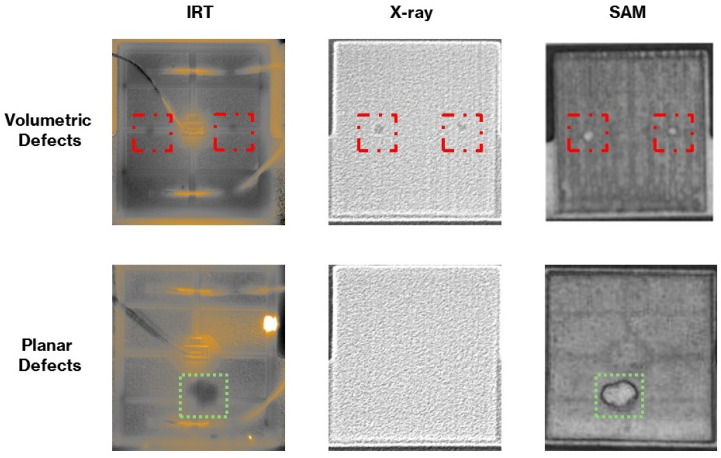
The inspection results of semiconductor packages in an IGBT module. Images on top indicate the presence of volume defect in the sintering layer, and those on the bottom reflect delamination at the sintering layer/die interface. Defects can be identified within the dashed boxes [12].

In Section 2.2, some renowned NDE/T methods for the inspection of microelectronic packages are introduced, together with a discussion of the drawbacks associated with each method. This section focuses on reviewing research works carried out on the nondestructive assessment of semiconductor packaging using SAM. Similar to other NDE/T methods, SAM has strengths and shortcomings, and the comparison between advantages of these methods for detecting anomalies in microelectronic packages has been the topic of study for several researchers. An example of such investigations can be found in the work of Dreher et al. [12], where the findings obtained from SAM were compared with X-ray and IRT images. According to their study, both the SAM and X-ray tests showed the presence of voids and volume flaws. However, the X-ray test was found to be less time-consuming. SAM’s primary benefit over X-rays lies in its ability to detect interface imperfections that X-rays may not be able to identify. Comparing the SAM and thermal results showed that although IRT images were capable of detecting both volumetric and zero-volume flaws, their resolution was much inferior to that of SAM C-scans. In Figure 9, the results of this experiment are provided. SAM’s superior performance compared to X-ray when inspecting large samples with high-density and fine interconnections has also been reported in the literature [92,93,94].

Knowing the advantages of SAM over other methods, the detailed studies are organized into three parts. First, publications focusing on defect detection are summarized. These works are grouped by the specific interface or structural level at which defects arise:

(i) Die-attach and sintered interfaces;
(ii) Bump/underfill solder interconnects;
(iii) Wire-bond interconnections and mold/leadframe interfaces;
(iv) TSVs and interposers;
(v) Cu–Cu (direct or hybrid) bonds.

This classification provides readers with a relative indication of where research efforts have been concentrated within the inspection of microelectronic packaging using SAM. Advances that improve SAM inspection outcomes are discussed in Section 4.2, followed by AI/ML approaches for automated damage assessment in Section 4.3.

### 4.1. Defect Identification

This subsection reviews research efforts that have utilized SAM for defect detection in semiconductor packages, with particular emphasis on the typical locations where defects form within microelectronic assemblies. Each study is briefly introduced in terms of its experimental focus, the types of defects investigated, and its key findings. To provide a clearer comparative perspective and highlight emerging trends in SAM applications, selected studies are further summarized, and associated challenges are critically analyzed.

#### 4.1.1. Die-Attach and Sintered Interfaces

Sintered silver and related die-attach layers in power modules are prone to voiding and interfacial degradation. SAM has been used to quantify porosity effects on acoustic response and wave velocity, and to distinguish induced flaws in Si-IGBT modules and bonded substrates [12,95]. The study by Dreher et al. [12] utilized SAM to detect and classify two distinct types of flaws occurring in Si-IGBT modules. The flaws were induced by selectively abrading a portion of the silver paste (the sintering paste) and chemically removing the metallic component of the chip. Gersh et al. proposed the use of multi-layer Direct Bonded Aluminium (DBA) as an alternative to Direct Bonded Copper (DBC) combined with silver sintering. SAM inspection was conducted to monitor the integrity of joint DBAs at various stages of cycling loads. The analysis determined that the suggested bonding technique exhibits fewer flaws and is more reliable.

#### 4.1.2. Bump/Underfill Solder Interconnects (Flip-Chip and Wafer-Level)

Underfill is a crucial aspect in enhancing the mechanical strength of solder-based packages, as it helps reduce the difference in thermal expansion coefficients between the die and substrate and it removes mechanical load from the solder joints [96]. Delamination in underfill can occur due to contamination, mechanical loads, and sudden temperature fluctuations, resulting in a stress fracture inside the bonding zone [97]. Xu et al. [98] conducted a study on the failure of adhesion in flip chip packages when subjected to thermal cycle loading. They utilized SAM C-scan images to accurately assess the cracks that developed within the underfill material.

In addition to underfill areas, solder bumps are another susceptible location where defects may form. Solder bumps can be as small as 30 μm and due to the growing need for miniaturizing the micro-packages, the size will likely shrink [99,100]. Constant or fluctuating temperature and the resulting stress variations (Thermo-mechanical failures) can be considered as the driving mechanism of crack formation and propagation in solder joints [101,102]. As the defects within the solder bumps are usually smaller than those found in the underfill, the first step in detecting solder damage is to distinguish the pulses received from the underfill and bumps. A variety of methodologies and techniques have been studied for this purpose, including the wavelet coefficient, the relative position of the signal peak, the width of the echoes, and the back-scatter Amplitude Integral. Brand et al. [92] reported that the peak amplitude of the echo in solder contact was 200% higher than that of underfill. Additionally, the pulse widths acquired from the solders and underfill were 30 and 10 ns, respectively. Having discerned the solder echoes, an operator can identify the defective regions by comparing the phase of the received signals, as is used in [103]. Some other studies have focused on extracting features from C-scan images as input to AI models, suggesting an automated approach for damage identification. These studies are discussed in Section 4.3.

#### 4.1.3. Wire-Bond Interconnections and Mold/Leadframe Interfaces

Wire bonding remains widely deployed across leadframe and laminate packages (e.g., DIP, QFP, QFN) [35,104]. A range of defects can appear during the manufacturing of wire-bonded packages; for instance, cracks, void formation, debonds at the package/leadframe and leadframe/chip interfaces, and lift-off [4]. SAM was used to detect defects commonly formed in these packages. Angelov et al. [105] utilized the concept of acoustic phase inversion in the presence of different mediums to identify poor bonding in encapsulation areas, copper pads, and pins. Mario and his colleagues [106] revealed in their study that high-frequency SAM has the capability to identify metal degradation and ball-bound defects in molded semiconductor packages.

#### 4.1.4. TSVs and Interposers

TSVs enable vertical routing but can suffer from incomplete fill, leading to the presence of incomplete penetration flaws. Zhang et al. [107] conducted a study to assess the capability of SAM in measuring the depth of TSVs utilizing a 110 MHz transducer. This study demonstrates the precise measurement (with less than 5% error) of TSVs at a depth greater than 30 μm utilizing the ToF concept. Grünwald et al. [108] performed a study on the automated failure analysis of 200 μm tungsten-coated TSVs with a depth of 100 μm using C-scan pictures. In this investigation, defective samples were created by subjecting them to electrical stress. Defective TSVs were identified by analyzing irregularities in the circular cross-section observed in the images. The primary focus of another contribution was the quantitative assessment of voids created in TSVs, as documented in the study of Kim et al. [109]. The study involved the detection of artificial voids with a size of 20 μm in area and 32.5 μm in depth using a transducer with a center frequency of 400 MHz.

#### 4.1.5. Cu–Cu Bonding (Direct and Hybrid)

TSVs and nano-TSVs were not the last advancements in reducing the interconnection pitch. Hybrid Cu-Cu bonding is a relatively advanced technology that offers wafer-level interconnection with a smaller pitch size. Void formation might occur during the annealing process in both the dielectric and copper joints. Detection of these flaws with SAM and evaluation of bond integrity have been investigated in the literature [110]. Gao et al. [111] explored the integration of direct bond interconnects in manufacturing environments using die-to-wafer or die-to-die assembly processes, focusing on topics such as Chemical Mechanical Polishing (CMP) extension to 10 μm pads, dicing, and surface preparation for bonding. Bond quality is evaluated using C-mode SAM, electrical resistance measurements, and cross-sectional microscopy, with the bonded parts showing enhanced reliability in standard thermal and high-temperature tests. Park et al. [112,113] developed a two-step Ar/$N_{2}$ plasma treatment to reduce the bonding temperature of copper. Using scanning acoustic tomography, they evaluated the bonding quality of this low-temperature Cu-Cu joint, demonstrating improved interface integrity and high shear strength. In another study conducted by Cho et al. [114], the impact of three different cleaning methods, namely, Ar sputtering in a vacuum, forming gas annealing, and $N_{2}$ annealing, on the defects at the interface of hybrid bonds was studied. The removal of the oxide layer to avoid flaw creation in copper bonding was also investigated by Hung et al. [115]. They recommended wet pre-treatment of the bonding surface with some acid solutions to remove the copper oxide layer. Similar to previous studies, a scanning acoustic technique was employed as an evaluation approach to assess bonding integrity.

Table 1 provides a summary of selected publications on the general usage of SAM for semiconductor packaging inspection.

The summarized studies in Table 1 collectively demonstrate the versatility of SAM as a nondestructive tool for defect characterization across a wide range of microelectronic package types. Conventional C- and B-scan modes, typically operating between 50 and 230 MHz, remain the most widely adopted configurations. These frequencies offer a practical compromise between penetration depth and lateral resolution, enabling the visualization of voids, delamination, and cracks within multilayer structures such as underfill, die-attach, and metallization interfaces.

However, the use of SAM in packages containing thick molding compounds may present challenges, as destructive operations are sometimes required to remove the mold material and expose the region of interest. In several studies, SAM has primarily served as a validation or complementary technique, for which experimental details are often limited. For instance, in [111,112,113], the center frequency of the employed transducer was not specified. Moreover, while some publications provide quantitative evaluations of the acquired results, a substantial portion of the reported work relies on qualitative comparisons. A deeper understanding of SAM’s capabilities and constraints will therefore require more systematic and quantitative assessments.

Furthermore, there remains a strong demand for higher inspection frequencies to detect smaller defects. As noted in [107,109], detecting voids smaller than approximately 30 μm necessitates the use of higher-frequency transducers that generate correspondingly shorter wavelengths. Nevertheless, due to the inherent trade-off between frequency and penetration depth, various remedies have been proposed to mitigate this limitation. These developments are discussed in the following section.

### 4.2. Resolution Enhancement

The continual decrease in the size of electronic packages results in a corresponding reduction in defect dimensions, making flaw detection increasingly challenging. Consequently, advanced NDE/T techniques are required to achieve higher sensitivity and spatial resolution. In immersion-based ultrasonic studies, such improvements can be pursued either by reducing noise in the received signals through signal processing techniques or by employing transducers with higher center frequencies, as discussed in Section 3.

**Figure 10:**
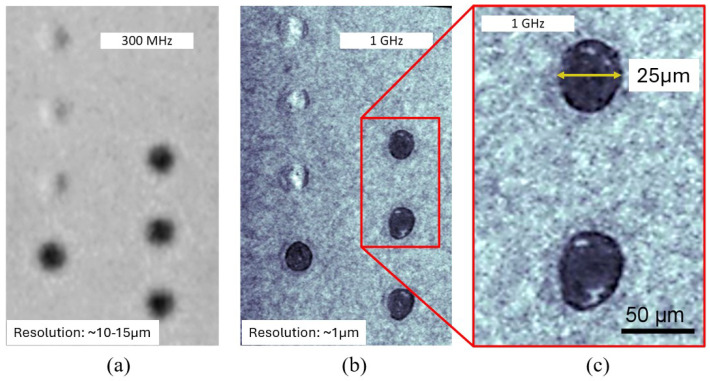
Evaluation of ball-bond integrity formed from a 25 μm Cu wire using two different acoustic transducers: (**a**) a conventional 300 MHz transducer with a 2.5 mm focal length (lateral resolution ∼ 10–15 μm); (**b**) an ultra-high-resolution 1.12 GHz transducer with an 80 μm focal length (lateral resolution ∼ 1 μm); (**c**) a magnified region from image (**b**) emphasizing the submicron feature definition achieved at gigahertz frequencies [120].

GHz-SAM has been studied as a means to enhance immersion ultrasound capabilities, and Brand and coworkers have been among the pioneers in extending the capabilities of acoustic microscopy into the gigahertz regime [116,117,118,119]. Their investigations demonstrated that using focused transducers operating up to 2 GHz enables the visualization of extremely small defects while also improving the temporal resolution of C-scan images, a crucial factor for analyzing miniaturized microelectronic structures. For instance, Vogg et al. [120] compared images of ball-bond and metallization regions acquired using a conventional 300 MHz transducer (lateral resolution of approximately 10–15 μm) with those obtained using a 1.12 GHz transducer (lateral resolution near 1 μm). As shown in Figure 10, the higher frequency and much shorter focal length (80 μm) produced a pronounced improvement in image clarity, enabling the visualization of fine interfacial details that were indistinguishable in the conventional setup.

The enhanced resolution provided by GHz-SAM is especially advantageous for inspecting stacked or heterogeneous assemblies such as 3D-ICs, for which conventional SAM systems yield limited information for structures below 15 μm [117]. In subsequent studies, Brand et al. [118] demonstrated the capability of GHz-SAM to detect both artificial and real TSV defects, including voids within via fillings (∼10 μm^3^) and rim delamination along via walls, by comparing C-scan and B-scan data acquired at 400 MHz and 1.2 GHz. Building upon these investigations, Brand et al. [121] further extended the use of GHz-SAM to chip-level metallization reliability studies. They applied 1 GHz acoustic microscopy, complemented by FIB/SEM validation, to analyze stress-induced voiding in AlCu power lines fabricated to emulate on-die interconnects of high-temperature ASICs. Voids smaller than the nominal acoustic resolution were successfully detected, and the introduction of a 40 nm TiN interlayer within the AlCu stack was shown to dramatically reduce void density. The study linked hydrogen-assisted embrittlement to Si_3_N_4_ passivation and highlighted TiN’s function as a hydrogen getter, thereby improving metallization reliability under thermal cycling. Collectively, these works established GHz-SAM as a benchmark for defect characterization across both wafer- and chip-level structures in microelectronic systems. The detection of nanometer-scale delamination in hybrid Cu–Cu/dielectric bonds used in wafer-to-wafer integration was investigated in [122]. Their experiments on patterned Cu pads with intentionally introduced air gaps revealed that GHz-SAM could resolve voids as thin as 100 nm and effectively distinguish bonded and unbonded regions.

While GHz-SAM provides outstanding resolution, the accompanying increase in frequency results in a significant rise in acoustic attenuation, and consequently, a reduced penetration depth [119]. This trade-off between resolution and depth often necessitates removing or thinning specific layers of the package. For example, Vogg et al. [120] applied plasma etching to remove the lead frame of plastic packages, allowing for direct access to the chip backside. Such requirements can partially compromise the nondestructive nature of SAM.

**Figure 11:**
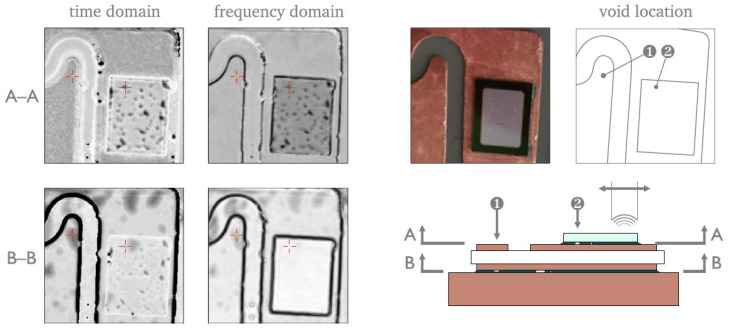
Comparison between time domain and frequency domain images obtained from testing a power module stack [125].

An alternative route to improving C-scan clarity, without increasing frequency or polishing samples, is through the use of advanced signal processing algorithms. These methods can enable the separation of overlaid echoes from different interfaces within micropackages [123]. For example, Jhang et al. [124] employed wavelet decomposition to separate echoes from the top and bottom surfaces of a die, and Uhrenfeldt et al. [125] used frequency-domain analysis to distinguish flaws at the chip/DBC and DBC/substrate interfaces (see Figure 11).

Brand et al. [126,127] employed a split-spectral processing technique to detect voids within 5 μm TSVs. Their results showed a substantial enhancement in image clarity compared to the raw data acquired using a 1 GHz transducer. This improvement stems from the method’s ability to selectively separate frequency components, thereby suppressing noise and improving contrast in high-frequency acoustic measurements. Such noise commonly originates from environmental disturbances, rapid attenuation of high-frequency components in GHz ultrasonic waves, or out-of-focus positioning of the transducer [128]. The study published by Su et al. [129] exemplifies the use of an optimal technique for reducing noise in high-frequency ultrasonic testing signals of flip chips. They achieved this by combining the Orthogonal Matching Pursuit method with an enhanced Artificial Bee Colony algorithm.

The advancement in enhancing the resolution and image quality of SAM is summarized in Table 2. The listed works collectively trace the evolution from early algorithmic processing methods to hardware-based GHz systems, as well as a combination of these two approaches. This overview provides a concise reference for assessing the overall progress and identifying the dominant trends in resolution-enhancement strategies.

The key findings of the summarized studies highlight the effectiveness of both data-processing algorithms and hardware advancements in enhancing acoustic image resolution. These improvements have significantly increased defect detectability, from the scale of tens of micrometers to only a few micrometers. However, such progress has come with notable trade-offs. In the case of hardware upgrades, particularly with the adoption of GHz-SAM, extensive surface preparation is often necessary to compensate for the limited penetration depth at higher frequencies. This necessity effectively changes SAM from a purely non-destructive to a semi-destructive method. Moreover, GHz operation introduces complex multimode propagation, making data interpretation considerably more challenging.

Conversely, signal- and image-processing techniques have primarily improved contrast and image clarity, but often at the expense of high computational costs. In most cases, these algorithms enhance image quality rather than substantially extending the probability of defect detection. A systematic study quantifying the Probability of Detection (PoD) for reference packages under different processing schemes could therefore provide a more rigorous basis for assessing the actual performance gain of various enhancement techniques.

A promising effort toward combining GHz-SAM hardware with signal-domain processing was reported in [126]. While this hybrid approach achieved superior resolution, it also introduced significant computational overhead. One practical strategy to mitigate this challenge is to restrict analysis to regions of interest (RoIs) and apply intensive algorithms locally rather than globally. The next section explores how artificial intelligence and machine learning methods are being leveraged to automate such RoI extraction and further accelerate acoustic microscopy data analysis.

### 4.3. Artificial Intelligence for Damage Detection

The manufacturing industry is currently undergoing substantial transformation under the influence of the fourth industrial revolution. A major objective of this shift is to enhance product quality while reducing human intervention [130,131]. In response, numerous studies have sought to develop intelligent and automated decision-making systems that minimize human dependency in quality control. Excessive reliance on operator experience often leads to subjective judgments and inconsistent evaluation of SAM images [132,133]. Consequently, a growing body of research has explored the application of machine learning and deep learning to enable automated flaw detection in SAM-based inspection.

One notable line of work focuses on feature-based classification using C-scan images. In this approach, RoIs are analyzed based on pixel brightness variations to extract descriptive features such as variance, skewness, and average gray level.

Su et al. [134] researched the automated detection of faulty solders in FC packages using a Support Vector Machine. Two samples with flip-chip interconnections were examined using a 230 MHz transducer, each containing a total of 317 microbumps. The study focused on analyzing the state of bumps at chip and substrate interfaces. The bumps in the C-scan pictures were designated as RoIs. In order to define the RoIs, a bump in the ideal state was manually chosen. Image extraction of each bump was accomplished by identifying its center using Normalized Cross Correlation and the size of the reference RoIs. The classification criteria were chosen as the aggregate gray value in images and the highest magnitude of signals associated with each bump at both interfaces. Furthermore, the contrast, determined by calculating the ratio between the highest amplitude at the bump-substrate interface and the highest amplitude at the chip-bump interface, was chosen as an additional characteristic.

Liu et al. [135] employed the Levenberg-Marquardt back-propagation network (LM-BP) to examine the categorization of faulty bumps in FC. LM-BP is an enhanced iteration of the BP neural network designed to address the limitations of the BP algorithm, including its restricted accuracy, low efficiency, and poor processing speed. The tested samples were fabricated using 135 μm bumps connecting dies to 635 μm thick FR-4 substrates. The RoIs were delineated by using binary masks of solder bumps generated based on the gradient of gray value in SAM pictures. The categorization criteria included in the study are variance, skewness, and average pixel intensity within the RoIs.

Several studies have leveraged SAM’s C-scan data for automated solder bump defect classification using diverse feature extraction and learning frameworks. A summary of key contributions is presented in Table 3. To maintain consistency across the reviewed studies, the notations for extracted features are standardized as follows: *A* and *I* represent the *area* and *summation of pixel intensity* (gray level) of a RoI, respectively. The subscripts *s* and *u* denote features corresponding to the solder and underfill regions. For instance, $A_{s}$, $A_{u}$, and $I_{RoI}$ refer to the area associated with solder, the area related to underfill, and the total intensity within a selected RoI, respectively. The symbol *V* denotes the average gray value per unit area (I/A), and *C* is defined as the contrast ratio between solder and underfill regions ($V_{s}/V_{u}$). In addition, *Var*, *Skew*, *Range*, and *Kurt* correspond to the variance, skewness, range, and kurtosis of pixel intensity distributions within each RoI. The acronym HOG appearing in Table 3 refers to the *Histogram of Oriented Gradients*.

Accurate segmentation and identification of RoIs remain a critical prerequisite for such analyses. Recent advances in deep learning segmentation frameworks have effectively addressed this challenge. A fine-tuned variant of the Segment Anything Model, termed SemiSA, has been developed for acoustic image segmentation [143]. Trained on more than 9000 CSAM images from diverse package types, SemiSA achieved Dice coefficients above 0.96 and up to 24% improvement in Intersection-over-Union over conventional networks. The study also released Ohlab-SemiSA, an open-source GUI for real-time SAM image analysis.

Although standard machine learning techniques are widely used, the process of feature selection can be time-consuming and demanding. Deep Learning algorithms are often suggested in the literature as a viable alternative due to their ability to do end-to-end learning and extract features directly from images. In [144], the authors employed Convolutional Neural Network models to estimate the porosity level in the Ag-sintered die-attaching layer, and the output of the network was compared with the porosity level estimated from the SEM images. Wang et al. implemented a solution by creating Very Deep Super Resolution (VDSR) images, as mentioned in their study [145]. VDSR stands for the utilization of a convolutional neural network to enhance picture resolution through the application of kernel filters of varying sizes across many layers.

The utilization of a physics-informed neural network framework to improve SAM images for advanced semiconductor packaging inspection is proposed by Ghosh et al. [146]. The model integrates acoustic wave propagation physics directly into the loss function, ensuring physically consistent image reconstructions while mitigating noise and improving spatial resolution. Their study on CoWoS-based GPU packages and flip-chip samples confirmed significant denoising and substantial compliance with acoustic physics, offering a foundation for future multimodal and real-time AI-driven inspection methods in heterogeneous packaging. Zhao et al. [147] employed the YOLOX architecture to investigate damage detection in plastic-encapsulated semiconductor packages. The YOLOX models were trained on 936 CSAM images, manually labeled to identify the location of delamination using square boxes. The results indicated that the YOLOX-X model with 640 × 640 input achieved the best performance with 95.5% mean Average Precision, efficiently identifying substrate, connecting-bar, and lead-wire delamination.

Beyond 2D C-scan data, one-dimensional waveforms and spectral features have been used for model training. As an example, authors in [148] used time and frequency data as the input of 1-D and 2-D CNNs to categorize the flip-chips into “defective Bumps”, “intact Bumps”, “delamination of underfill”, and “background/undefined” classes. Nair et al. [149] extended this direction by directly applying deep networks to A-scan signals. A wavelet filter was applied for denoising and normalization, and A-scan signals were labeled through a semi-supervised expert-assisted process. After wavelet-based denoising and semi-supervised labeling, architectures including CNNs, RNNs, and hybrid CNN–RNN models were evaluated. The authors highlighted two advantages of waveform-based learning: minimal labeling effort and improved detection of low-contrast defects invisible in C-scan images.

Despite these advances, most deep learning studies continue to face the persistent challenge of limited datasets [150]. Common remedies include data augmentation, transfer learning, and metric learning [151,152,153]. Among these, data augmentation helps mitigate overfitting by expanding datasets through geometric or noise-based transformations [154,155]. In studies focusing on SAM and microelectronic packaging, Sha et al. [156] proposed a Residual Attention Generative Adversarial Network (RA-GAN) to address data scarcity in flip-chip inspection using SAM. The model integrated a residual attention module into the GAN architecture to enlarge the perceptual field and better capture fine structural details in the generated images. Trained on a limited set of SAM images, RA-GAN produced synthetic, high-quality, and diverse samples that effectively expanded the dataset. These augmented data were then used to train deep learning classifiers, leading to improved accuracy and robustness in defect recognition. This work highlights how GAN-based augmentation can effectively alleviate data scarcity in SAM applications, enhancing model generalization when large experimental datasets are impractical. Notably, combining RA-GAN with *ResNet18* achieved the highest defect classification accuracy of 99.71%. Another GAN model for addressing the data scarcity in training Neural Networks (NNs) was proposed in [157]. The study combined synthetic data generation, transfer learning, and super-resolution to enhance SAM image quality and reduce scanning time. A high-order degradation model was developed to generate realistic low-resolution synthetic datasets from HR images, simulating real scanning artifacts such as blur, noise, and compression. These synthetic data were then combined with real SAM images to train the GAN model. Transfer learning from the *DIV2K* dataset further improved model convergence and generalization on limited industrial data. Quantitative evaluations showed that the proposed GAN achieved an average PSNR of 33.98 dB and SSIM of 0.91, outperforming other state-of-the-art models.

Finally, since SAM images of damaged samples often exhibit poor contrast, future efforts may benefit from anomaly detection frameworks, where models are trained exclusively on defect-free data and deviations are flagged as potential damage. Such strategies could enable reliable defect detection even when labeled defect data are scarce.

While considerable progress has been made, as summarized above, several practical issues remain when using SAM for evaluating the structural integrity of microelectronic packages. Among all these challenges, the resolution–depth trade-off at higher frequencies (often necessitating layer thinning that renders inspections semi-destructive), complex multimode propagation at GHz frequencies that complicates signal interpretation, and echo overlap in thin multilayer stacks are notable. Advanced processing pipelines further introduce substantial data volumes and computational cost, and much of the literature still lacks systematic, quantitative benchmarking (e.g., Probability of Detection) for fair comparison across package types and defects. Operational constraints (immersion coupling and cycle time) also limit in-line use. Finally, deep-learning approaches face data scarcity, which this review notes has been mitigated through data augmentation, transfer learning, and GAN-based synthesis; however, the broader availability of curated datasets is still needed. Partial remedies discussed in Section 4.2 and Section 4.3 include wavelet, frequency-domain, split-spectral processing for echo separation, restricting computation to RoIs (with automated RoI extraction), and hybrid GHz-SAM; nevertheless, scalable implementations and standardized metrics remain open to investigation.

## 5. Future Works

Building upon the current challenges identified in the application of SAM for integrity assessment of semiconductor packages, as well as general necessities in the field of NDE/T, several prospective research directions and development needs have been identified:

- *Hardware Upgrade:* Increasing the center frequency of transducers inherently reduces penetration depth, yet this approach remains essential given the continual miniaturization of modern microelectronic components. To alleviate this trade-off, the introduction of advanced excitation schemes, such as chirp waveforms, narrow-band pulses, or tone bursts with adjustable cycles, could enhance defect detectability and extend the sensitivity range of SAM. Implementing such waveform variations will necessitate corresponding upgrades in the signal generation, transducer design, and receiver electronics of SAM systems. These improvements could also facilitate compatibility with coded-excitation and pulse-compression techniques, paving the way for higher signal-to-noise ratios without compromising imaging depth.
- *Signal and Data Processing:* Future research should emphasize data-processing strategies that go beyond enhancing image contrast to directly improving defect detectability. Advanced beamforming and aperture-focusing algorithms could help reduce the number of scans required for layer-by-layer evaluation, thereby accelerating inspections while mitigating issues associated with overlapping signals. Moreover, physics-informed modeling and simulation frameworks can be leveraged to generate high-fidelity synthetic data for algorithm training, addressing data scarcity in experimental measurements. The accuracy of such models will be critical for ensuring that simulation-based analyses faithfully capture the acoustic behavior of multilayer microelectronic structures. Finally, scalable processing pipelines should be developed to minimize computational overhead while maintaining quantitative accuracy.
- *Robotics and Automation:* The integration of cutting-edge robotic platforms can substantially enhance the speed, precision, and reliability of SAM-based inspections. Autonomous and collaborative robots are increasingly deployed in semiconductor fabs to handle complex, high-throughput tasks within cleanroom environments. Extending this capability to NDE/T, particularly SAM, offers clear potential for automating sample handling, probe positioning, inference, and RoI extraction, reducing manual intervention and improving measurement consistency. Two practical considerations motivate this direction: (i) GHz-SAM systems operate with extremely short working distances; so, manual adjustments of the water path or focal plane carry a nontrivial risk of collision, potentially damaging either the transducer or the sample; and (ii) even slight angling introduced during sample mounting (e.g., trays screwed into the tank base) or during transducer positioning can deviate from perpendicular incidence, strengthening unintended ultrasonic modes and exacerbating multimode interpretation at high frequencies. Robotic assistance that maintains precise standoff and alignment, with closed-loop feedback, can mitigate these risks while enabling repeatable, high-quality inspections.
- *Digital Twin and NDT 4.0:* When thinking about additional advancements for the SAM technology, the use of Digital Twins and the NDT 4.0 should be considered. Regarding SAM and NDE 4.0, the continuous alignment of virtual process models with actual inspection data would empower predictive maintenance, remote monitoring, and closed-loop optimization of inspection parameters. SAM in a digital-twin ecosystem enables real-time decision-making and provides traceable inspection records, which facilitate accountable design, fabrication, and reliability feedback loops for advanced microelectronic packaging.
- *Workforce Development:* The microelectronics manufacturing industry is facing a significant workforce shortage. For instance, in the U.S., this shortage is projected to exceed 27,000 by 2030, including 5300 PhDs, 12,300 Master’s, and 9900 bachelor’s degree holders [158]. Addressing this shortage requires sustained effort and innovative approaches to education and training.

By focusing on these areas, the research community can significantly advance the capabilities of NDT including SAM technology in semiconductor and microelectronics manufacturing, ensuring higher quality and reliability in the production of future technologies.

## 6. Conclusions

The significance of microelectronic packages in the electronics industry cannot be overstated, as they provide essential protection for semiconductor chips that form the backbone of a wide range of devices, from mobile phones to advanced computing systems. As technology continues to advance, the reliability and functionality of these devices increasingly rely on the effectiveness of inspection methodologies. Nondestructive Evaluation (NDE/T), including Scanning Acoustic Microscopy (SAM), has emerged as a crucial technique for the comprehensive inspection of these packages. It enables damage-free assessments that ensure both quality assurance and the advancement of resilient, dependable electronics to meet present and future requirements. This scholarly review has thoroughly evaluated the progress and application of SAM for assessing the structural integrity of microelectronic packages. Our analysis focuses on SAM, highlighting its essential role among various NDE/T methodologies, especially in addressing the challenges posed by the increasing complexity and miniaturization of microelectronic devices. The unmatched ability of SAM to provide high-resolution imagery is crucial for the early identification of potential failures, a critical aspect in ensuring the reliability and performance of semiconductor devices. Throughout this examination, we have explored numerous significant technological advancements in SAM, emphasizing its integration with artificial intelligence and advanced signal processing techniques, which enhance the capabilities for defect detection and characterization. Despite these technological advancements, this review points out several ongoing challenges, particularly regarding limitations in lateral and axial resolution, as well as the need for improved penetration depth without compromising resolution. Looking forward, the integration of SAM with emerging technologies such as AI, machine learning and automated image analysis, robotics, and digital twin, holds promise for further enhancing its capabilities and applications. Additionally, the development of transducers with higher frequencies and application-adjusted apertures could open up new possibilities in microelectronic inspection, enabling more detailed and precise assessments.

## Figures and Tables

**Table 1:** Summary of conventional SAM-based studies on inspection of microelectronic packages.

Year	Package Type	Inspection Location/Region	Defect Type	Key Findings	Limitations/Challenges	Ref.
2000	Flip-chip	Underfill/chip interface	Delamination	Used 230 MHz transducer to detect controlled delamination before and after thermal cycling in C-scan images	Could not reveal poor adhesion in the absence of air gaps; failed to detect weak bonding without separation	[96]
2004	Flip-chip	Underfill/chip interface	Delamination	Measured delamination crack length and growth rate under thermal cycling using C-scans of a 230 MHz transducer; correlated with FE J-integral to derive a Paris-type reliability relation	No specific SAM-related limitations reported	[98]
2016	Flip-chip	Die/solder bump interface	Micro-crack	The B-scan and C-scan pixel-intensities of a 230 MHz transducer used as features for an indirect estimation of crack growth under thermal cycling; validated via simulation and accelerated testing	Quantitative accuracy limited by transducer resolution and edge-effect interference; mainly applicable to cracks near bump periphery	[99]
2018	Flip-chip	Underfill and interconnect regions	Internal voids, mold voids, delamination	Demonstrated feasibility of 50 MHz transducer C-scan images for mold void detection; SAM indications validated destructively	Verification still required destructive tests	[103]
2010	3D IC	TSV depth and wafer interfaces	Depth variation and incomplete etching	A-scans were used to extract time-of-flight to measure TSV depth using a 110 MHz transducer; B-scans identified via depths with <5% error vs cross-section; effective for vias ≥ 30 μm	Accuracy limited for smaller vias (<30 μm) due to diffraction and resolution; higher-frequency transducers recommended	[107]
2013	3D IC	Cu-Cu direct bonding interface	Interfacial voids from oxide and stress non-uniformity	Studied surface cleaning effects on Cu–Cu bonding; C-scans were used to localize voids	Frequency unspecified	[114]
2021	3D IC	Cu-Cu hybrid bonding interface	Voids; oxidation-induced bonding failures	C-scans used for evaluating wet-acid pretreatments (acetic, citric, sulfuric, hydrochloric) for oxide removal prior to hybrid bonding	Frequency unspecified	[115]
2023	3D IC	TSV	Internal voids and seam defects in via holes	Custom ultra-high-resolution 400 MHz SAM detected 20 μm voids at ∼32.5 μm depth (confirmed by FIB-SEM); precise visualization of TSV defects through C-scans and B-scans	Detection limited by scattering at extreme depths; further hardware optimization needed for vias < 20 μm	[109]
2016	Bonded wafers	TSV and Si substrate	Crack in TSV coating; defect at TSV bottom	Detected damage of ∼50 μm with a 100 MHz transducer; SAM’s C-scans were validated with X-ray; provided EFIT simulation	No specific SAM-related limitations reported	[108]
2018	Die-to-wafer and die-to-die bonding	Cu–Cu bonding interface	Voids/unbonded regions	CSAM assessed Cu-Cu bond quality; SAM-identified voids confirmed by electrical testing	Frequency unspecified	[111]
2019	Bonded wafers	Cu-Cu bonding interface	Incomplete Cu diffusion/poor bonding	CSAM (SAT) provided qualitative evaluation of Cu-Cu interface after plasma treatment	Frequency unspecified	[113]
2021	Bonded wafers	Cu-Cu bonding interface	Unbonded/void regions	CSAM (SAT) localized unbonded regions attributed to SiO_2_ patterning and etching defects	Frequency unspecified	[112]
2012	Power semiconductor device (DIP-style)	Die-attach and wire-bond interfaces	Delamination, voids, bond lift-off	Performed inspection of power devices using CSAM (75 and 230 MHz)	Inspections required metallization removal via mechanical grinding and chemical etching	[106]
2018	Si-die on DCB substrate	Die-attach (Ag-sinter layer)	Porosity (1–5 μm pores)	Correlated 175 MHz SAM B-scans (reflectivity + ToF) with porosity level	Quantitative porosity estimation limited by sinter-layer thickness variation and scattering from other inhomogeneities	[95]
2018	Si-IGBT on DBC substrate	Die-attach (Ag-sinter joint)	Voids; adhesion defects (interface delamination)	Minimum detectable defect reported as ∼427 μm (circular) or ≥120 μm (line); interface defect >451 μm for a 50 MHz transducer; sensitive to porosity	Requires water coupling; inspection time ∼8 min per substrate; in-line integration limited by immersion setup	[12]

**Table 2:** Summary of studies focused on resolution enhancement and advanced signal or image processing in SAM for microelectronic inspection.

Year	Technique	Enhancement Strategy	Key Findings	Limitations/Challenges	Ref.
2011	GHz-SAM	1 GHz transducer (burst mode)	Achieved ∼3 μm resolution through ∼5 μm polymer; localized delaminations in Cu/Sn micro-bump arrays verified by SEM	Limited penetration depth; requires thinning and surface access	[116]
2014	GHz-SAM	1.12 GHz for 3D IC applications; focus and defocus imaging for near-surface defect localization	Demonstrated the superiority of GHz-SAM in inspection of TSVs smaller than 100 μm over a 200 MHz transducer; GHz-SAM lateral resolution was about 1–3 μm; detected voids ∼1.5 μm below the surface; verified acoustic contrast by PFIB and SEM	Limited penetration depth (<10 μm); sensitivity to surface condition	[117]
2015	GHz-SAM	1.12 GHz for 3D IC applications	Provided comparative analysis between 400 MHz and 1.12 GHz; demonstrated that 1.12 GHz SAM achieved ∼1–3 μm lateral resolution, enabling detection of voids and rim-delaminations at depths up to ∼40 μm; 400 MHz scans provided lower resolution but deeper penetration; verified structural correspondence with FIB/SEM	Penetration depth limited (<50 μm); interpretation complicated by multimode propagation	[118]
2015	GHz-SAM	1 GHz for inspection of molded packages	A Comparative analysis between several MHz range transducers and the GHz transducer is provided; The employed transducers were varied with respect to lens aperture; Adhesion defects and micro-voids in 25 μm Cu ball bonds and 30 μm wire–metal contacts were clearly shown; emphasized on the necessity of GHz-SAM for reliable evaluation of 10 μm	Limited penetration depth at 1 GHz (<10 μm); requires thinning and surface access	[120]
2016	GHz-SAM	1 GHz for inspection of AlCu power lines	Stress-induced voids in the power lines were inspected using GHz-SAM; verification of the results was performed with FIB/SEM cross-sections	Limited to near-surface inspection (<10 μm depth); requires thinning and surface access; quantitative depth estimation complicated by varying acoustic velocities.	[121]
2007	Signal Processing	Sparse signal representation and adaptive dictionary learning	Introduced a signal-processing framework using matching-pursuit and adaptive sparse representations in Gabor dictionaries to decompose ultrasonic A-scans; enabled separation of overlapping echoes; improved time-of-flight accuracy; enhanced lateral and depth resolution beyond conventional 230 MHz SAM performance	Computationally expensive; sensitive to noise model and sample-dependent echo distortions	[123]
2011	Image Processing	Blind deconvolution; Gaussian and Sobel filters	Enhanced edge definition and contrast in bonded-wafer and interfacial images	Parameter tuning required; processing not standardized across samples; improvement primarily near-surface	[116]
2011	Signal Processing	Wavelet-based features; backscatter amplitude integral (BAI); parametric/cepstral analysis for echo separation	Improved defect detectability and interpretability in inspection of flip-chip interconnections; Better adhesion contrast compared to C-scans of raw data; Verification performed with SEM and X-ray microscopy	Computationally intensive; method and thresholds must be re-tuned for device regions and stacks; sensitivity to noise/sampling	[116]
2018	Signal Processing	Frequency-domain transformation	Introduced a frequency-domain SAM framework for multilayered power-module inspection; Fourier analysis of gated A-scans enabled layer-selective contrast and thickness estimation by identifying resonant dips and harmonic interference frequencies linked to internal reflections; demonstrated accurate separation of overlapping echoes	Analysis limited to 25 MHz transducer range	[125]
2018	GHz-SAM and Signal Processing	1 GHz transducer and FFT-based computation of power spectral density	Developed time-resolved acoustic gigahertz microscopy (GHz-SAM) combined with spectral-domain analysis of unprocessed RF echo data for inspection of TSVs; power spectral density computed in 5 MHz bands (800–1200 MHz) enabled frequency-selective imaging and improved defect sensitivity; the method successfully detected sub-surface voids (∼3–10 μm) in Cu-filled TSVs; validated with FIB/SEM; demonstrated that spectral decomposition increases sensitivity to weak scattering signals compared to intensity-only SAM.	Limited penetration depth (<10 μm); high-frequency attenuation; Large dataset and computationally expensive	[126]
2020, 2023	Signal Processing	Sparse signal reconstruction	Introduced sparse-representation denoising using Gabor dictionaries and orthogonal matching pursuit ([128]), later refined with adaptive artificial-bee-colony optimization (AABC-OMP) and wavelet-threshold post-processing ([129]). The approach separates overlapping echoes, enhances SNR, and improves convergence in high-frequency SAM of flip-chip solder joints	Computationally expensive	[128,129]

**Table 3:** Summary of studies on automated damage identification in flip chips using SAM C-scan images.

RoI Extraction	Features	Classification Method	Ref.
Cross-Correlation	$A_{s}$, $A_{u}$, $I_{s}$, $I_{RoI}$, $V_{s}$, $V_{u}$, *C*	BP Network	[136]
	$A_{s}$, Var, Kurt	FCM	[137]
Binary Mask	$A_{s}$, Mean, Range, Var, Skew	Fuzzy-SVM	[138]
	Var, Mean, Skew	Radial Basis Function Neural Network	[139]
Correlation Coefficient	$A_{s}$, Mean, Var, Range	General Regression Neural Network	[140]
HOG	$A_{s}$, Mean, Range	SVM	[141]
Threshold Gradient	$A_{s}$, $A_{u}$, $I_{s}$, $I_{u}$, $V_{s}$, $V_{u}$, *C*	Improved Decision Tree	[142]

## Abbreviations

The following abbreviations are used in this manuscript:

**3D-IC**	Three-Dimensional Integrated Circuit
**Ag**	Silver
**Au**	Gold
**BCIT**	Barker Code Infrared Thermography
**BGA**	Ball Grid Array
**BP**	Back Propagation
**CMP**	Chemical Mechanical Polishing
**Cu**	Copper
**DBA**	Direct Bonded Aluminium
**DBC**	Direct Bonded Copper
**DBSCAN**	Density-based Spatial Clustering of Applications with Noise
**DIC**	Digital Image Correlation
**ECPT**	Eddy Current Pulse Thermography
**EM**	Electromigration
**FC**	Flip-Chip
**FCM**	Fuzzy C-means clustering
**HOG**	Histogram of Oriented Gradient
**I/O**	Input/Output
**IC**	Integrated Circuit
**IMC**	Intermetallic Compounds
**IRT**	Infrared Thermography
**LFMTWI**	Linear Frequency Modulation ThermalWave Imaging
**LIT**	Lock-in Infrared Thermography
**LM-BP**	Levenberg-Marquardt Back Propagation
**LUT**	Laser Ultrasonic
**NDE/T**	Nondestructive Evaluation/Testing
**NDT**	Nondestructive Test
**RoI**	Region of Interest
**SAM**	Scanning Acoustic Microscope
**SiP**	ystem-in-Pakcage
**SNR**	Signal-to-Noise Ratio
**TAB**	Tape Automated Bonding
**ToF**	Time-of-Flight
**TSV**	Through Silicon Vias
**UBM**	Under Bump Metallization
**UT**	Ultrasonic Test
**VDSR**	Very Deep Super Resolution
**WB**	Wire Bonding
**XCT**	X-ray Computed Tomography
**XRM**	X-ray Microscopy
